# MiR-489 aggravates H_2_O_2_-induced apoptosis of cardiomyocytes via inhibiting IGF1

**DOI:** 10.1042/BSR20193995

**Published:** 2020-09-16

**Authors:** Shan Tang, Hongyan Zhong, Ting Xiong, Xinquan Yang, Yongqing Mao, Daxin Wang

**Affiliations:** 1 The Second Xiangya Hospital of Central South University, Changsha, China; 2 Clinical Medical College, Yangzhou University, Yangzhou, China

**Keywords:** bioinformatics, H9c2, IGF1, miR-489, Myocardial infarction

## Abstract

Myocardial infarction (MI) is a major type of cardiovascular disorder worldwide. In the present study, we established a new microRNA (miRNA)–mRNA cross-talk network by integrating data obtained from The National Center for Biotechnology Information Gene Expression Omnibus (NCBI GEO). In addition, functional assays, including Kyoto Encyclopedia of Genes and Genomes (KEGG) pathway and Gene Ontology (GO) analyses, were conducted using the Database for Annotation, Visualization, and Integration Discovery (DAVID). In our study, we generated a new differentially expressed miRNA (DEmiRNA)-differentially expressed gene (DEG) cross-talk network of MI composed of three miRNA (miR-489, miR-375, and miR-142-3p) nodes and 163 mRNA nodes. *In vitro* experiments demonstrated that miR-489 expression was increased in H_2_O_2_-treated H9c2 cardiomyocytes *in vitro*, mimicking myocardial injury. We observed that down-regulation of miR-489 reduced H_2_O_2_-induced apoptosis, while overexpression of miR-489 had the opposite effects, as revealed by flow cytometry and Western blot analyses. Furthermore, we confirmed the relationship between miR-489 and IGF1 through double luciferase reporter gene assays, which partly explains the antiapoptotic mechanism of miR-489. In conclusion, the experimental results of the present study could provide important clues for investigating the mechanism of MI.

## Introduction

Myocardial infarction (MI) is a common and catastrophic cardiovascular disorder characterized by myocardial necrosis induced by long-term ischemia [1]. Acute MI (AMI) is the primary cause of cardiovascular disorders and is a highly common cause of mortality and disability [2]. Nearly 550,000 first-episode and 200,000 recurrent AMIs occur each year, causing major social and economic burdens on healthcare systems [3]. In the clinic, the diagnosis and treatment of AMI are primarily based on clinical symptoms.

At present, the clinical treatment of acute myocardial infarction primarily involves the recanalization of large blood vessels, which results in myocardial ischemia–reperfusion injury. There has been a great deal of work to elucidate the cause of such injuries, but reperfusion injury is still unavoidable once revascularization is performed [4–6]. Currently, although extensive efforts have been made to elucidate the molecular mechanisms that contribute to AMI [7–10], the etiology and pathogenesis of this condition remain unclear. Therefore, there is an urgent need to identify biomarkers and mechanisms for this condition for the prediction and treatment of MI.

MicroRNAs (miRNAs) are a type of small noncoding RNAs with fewer than 22 nucleotides that negatively regulate transcription by inhibiting protein translation or degradation. Increasing numbers of studies have shown that abnormal miRNA expression can lead to various cardiovascular disorders, including cardiac ischemia and vascular atherosclerosis [11,12]. For example, miR-342-5p in circulating exosomes induced by long-term exercise can have an endogenous cardioprotective role in myocardial ischemia/reperfusion injury by inhibiting hypoxia/reoxygenation-induced cardiomyocyte apoptosis by targeting caspase 9 and Jnk2 and activating p-Akt signaling through regulation of Ppm1f [13].

The role of bioarray techniques in basic research has been increasingly recognized by scientists, as it allows for the identification of new genes and noncoding RNAs linked to multiple diseases, providing potential clues and scientific evidence for further studies using bioinformatics tools [14]. In the present study, the gene expression profile of GSE34198 and the miRNA expression profiles of GSE61741 and GSE31568 were obtained from the Gene Expression Omnibus (GEO) [15–17]. The limma package and metaMA package in R were used to identify differentially expressed (DE) genes (DEGs) and DE miRNAs (DEmiRNAs) of MI, respectively. Enrichment analyses were conducted to elucidate the functions of the DEGs in MI. We constructed a protein–protein interaction (PPI) network of the DEGs and defined the genes with a high values in the network as hub genes. We predicted the target genes of DEmiRNAs by searching the miRWalk database and establishing a new miRNA–mRNA regulatory network that is involved in the development and occurrence of MI; these findings will provide new insights for the diagnosis and treatment of MI.

We identified several DEmiRNAs associated with MI, including miR-489, miR-1274b, miR-142-3p, and miR-375. The results of a previous study proved that inhibition of both endothelial miR-92a and miR-489 can reduce chronic kidney disease-associated atherosclerosis, repressing the elevated expression of Fam220a and Tgfb2, respectively [18]. In a rat model of gentamicin injury, elevation of miR-489 in urine preceded elevation of urinary creatinine and blood urea nitrogen, suggesting that urinary miR-489 could be a new biomarker of kidney injury [19]. Interestingly, intrarenal miR-489 and urinary miR-489 were both elevated in a kidney model of ischemia–reperfusion injury [20]. Previous research has shown that extracellular and cellular levels of miR-489-3p are related to cardiovascular and kidney disorders, but the function, mechanism, and therapeutic potential of miR-489-3p in MI have not been reported. Therefore, the present study, we generated an *in vitro* MI model using H9c2 cardiomyocytes to assess MI and verify the effects of miR-489 in myocardial damage.

## Materials and methods

### Gene expression profile data

The gene expression profiles of GSE34198 and the miRNA expression profiles of GSE61741 and GSE31568 were obtained from the GEO database. In these GSEs, 10 samples were randomly selected for differential expression analysis.

### Screening for DEGs and DEmiRNAs

The DEGs were identified using an empirical Bayesian method through the R “limma” package. The threshold of DEGs was defined with |logFC| > 1 and *P*-value < 0.05. In addition, the metaMA package, which can handle missing data and eliminate batch effects, was used to integrate the different platforms [21]. We used the limma and metaMA packages to confirm the DEmiRNAs, and |ES| > 6 and FDR < 0.05 was used to filter the identified DEmiRNAs.

### Functional enrichment analysis of DEGs

To annotate the genes and identify characteristic biological attributes for these DEGs, we performed Kyoto Encyclopedia of Genes and Genomes (KEGG) pathway and Gene Ontology (GO) analyses with the Database for Annotation, Visualization and Integrated Discovery (DAVID). Each item of enrichment had a cut-off criterion of *P*<0.05.

### Construction of a PPI network

To uncover the core regulatory genes, we used the STRING database [22] to construct a PPI network. These interaction networks were visualized using Cytoscape [23].

### Prediction of target genes of DEmiRNAs

To predict targets of DEmiRNAs, we used the online database miRWalk. In addition, to screen for predicted target genes, we constructed a regulatory network between DEmiRNAs and DEGs.

### H9c2 cell culture and treatment

Mouse H9c2 cardiomyocytes (Chinese Academy of Sciences, Shanghai, China) were cultured at 37°C in a humidified incubator under an atmosphere with 5% CO2 in Dulbecco’s modified Eagle’s medium supplemented with 10% fetal bovine serum (Gibco, U.S.A.), 100 units/ml penicillin, and 100 μg/ml streptomycin (Suo Lai Bao Biotechnology Co., Ltd., Beijing, China). To establish the *in vitro* model of MI, we seeded cells in six-well plates at a density of 1 × 10^5^ cells/well and treated them with 0, 100, 200, 400, 450, and 500 μM H_2_O_2_ for 1 h, with 450 μm selected for analysis in the subsequent experiments.

### H9c2 cell transfection

To perform gain- and loss-of-function analyses, we seeded H9c2 cells in six-well plates and performed transfections when the cells are at logarithmic growth period. In addition, under good cell growth conditions, the cells exhibited a strong refractive property, a plump cytoplasm and a clear nuclear cytoplasm under a microscope. The cells were transfected with 50 nM miR-489 mimic and 100 nM miR-489 inhibitor or miRNA-inhibitor negative control (NC) (RiboBio, China) using RiboFECT™ CP reagent (RiboBio, China) following the manufacturer’s instructions. Protein and RNA extraction was performed after 48 h.

### Cell Counting Kit-8 (CCK8) assays

Cell viability was examined by CCK8 (Dojindo, Japan) assays. We seeded 100 μl of cell suspension per well into 96-well plates at a density of 5000 cells/well. At specific time points, we added 10 μl of CCK-8 solution to the cells and incubated the samples for 2 h at 37°C. Then, the reaction product was quantified following the manufacturer’s instructions.

### Western blot analysis

H9c2 cell extracts were prepared in RIPA lysis buffer (Suo Lai Bao, Beijing, China) containing 1% phenylmethanesulfonyl fluoride (PMSF). Total proteins were quantified with a BCA protein assay kit (Vazyme, China). The proteins were electrophoresed in sodium dodecyl sulfate (SDS)-polyacrylamide gels and then transferred to PVDF membranes (Millipore, MA, U.S.A.). The membranes were incubated for 2 h with 5% nonfat milk in Tris-buffered saline for blocking. Subsequently, the membranes were incubated overnight at 4°C with the following primary antibodies: anti -caspase-3 (1:1000, Proteintech, 19677-1-AP), anti-Bcl-2 (1:1000, Proteintech, 26593-1-AP), anti-BAX (1:1000, Proteintech, 50599-2-Ig), anti-IGF1 (1:1000, Abclonal, Huohao), and anti-β-actin (1:5000, Sigma, A2228). Then, the membranes were incubated with the appropriate secondary antibodies for 2 h on a shaker at room temperature. The protein levels were quantified by relative densitometry and normalized to that of β-actin as an internal control.

### RNA extraction and RT-qPCR

The miR-489 levels in H9c2 cells were measured using RT-qPCR. After H_2_O_2_ treatment, the total RNA of the samples was extracted using TRIzol reagent (Tiangen, China), after which 1 μg of RNA was used to synthesize cDNA using a RevertAid First Strand cDNA Synthesis kit (Thermo Fisher Scientific, U.S.A.). Gene expression was assessed with Genious 2× SYBR Green Fast qPCR Mix (High ROX Premixed) (ABclonal, China).

MiR-489 primers: F: 5′-CCGCCATGACATCACATATATG-3′, R: 5′-CAGTG CGTGTCGTGGAGT-3′, and RT: 5′-GTCGTATCCAGTGCGTGTCGTGGAGTC GGCAATTGCACTGGATACGACGCTGCCA-3′; and U6 primers: F: 5′-CTCG CTTCGGCAGCACA-3′, R: 5′-AACGCTTCACGAATTTGCGT-3′ and RT: 5′- AACGCTTCACGAATTTGCGT-3′.

### TUNEL assay

The apoptosis of H9c2 cells was assessed using a one-step TUNEL assay kit (KeyGen, Nanjing, China). After being fixed with 2% formaldehyde and permeabilized on ice with 0.1% Triton X-100, samples were then incubated at 37°C with TUNEL reaction buffer according to the manufacturer’s protocol. Subsequently, fluorescence microscopy was used to observe the apoptotic cells.

### Cell apoptosis analysis

The apoptosis rate of cells was evaluated using an Apoptosis Detection Kit (Vazyme Biotech) following the manufacturer’s instructions. The apoptotic rate was obtained by calculating the sum of the ratio of the right upper quadrant and the right lower quadrant.

### Double luciferase reporter gene assay

The binding domain of miR-489 and the 3′ untranslated region (UTR) of IGF1 was obtained from the online database miRWalk. The 3′-UTRs of IGF1 with wild-type and mutant binding sites for miR-489 were generated by RiboBio (Guangzhou, China) and cloned into pmiR-RB-REPORT (RiboBio). The vectors were cotransfected with miR-489 mimic and control into 293T cells, after which the cells were incubated for 48 h and then analyzed for luciferase activity. Next, the Dual-Glo® Luciferase Assay System (Promega, Madison, U.S.A.) was used to calculate the relative luciferase activity following the manufacturer’s protocol

### Statistical analysis

The results are presented as the means ± SEM. Statistical comparisons among different groups were conducted by one-way ANOVA, while differences between two groups were assessed by Student’s t-test. *P*<0.05 was defined as significant.

## Results

### Screening for DEGs

We identified 688 DEGs (including 500 up-regulated genes and 188 down-regulated genes) from the gene expression datasets (criteria of *P*<0.05 and |logFC| ≥ 1). In addition, we obtained four DEmiRNAs based on |combined ES| > 6 and FDR < 0.05 using the metaMA package and the miRNA expression dataset. The top 10 DEGs and DEmiRNAs are shown in Supplementary Tables S1 and S2, respectively.

### Functional analysis results

DAVID was used for GO functional analysis and KEGG pathway enrichment analysis. The GO analysis results indicated that the DEGs were primarily enriched in “histone acetyltransferase activity”, “positive regulation of transcription from RNA polymerase II promoter”, and “signal transduction involved in regulation of gene expression” (Supplementary Table S3). The primary KEGG pathways were “Cytokine–cytokine receptor interaction”, “Glycosphingolipid biosynthesis - lacto and neolacto series”, and “Intestinal immune network for IgA production” (Supplementary Table S3).

### Hub genes in the PPI network

**Figure 1:**
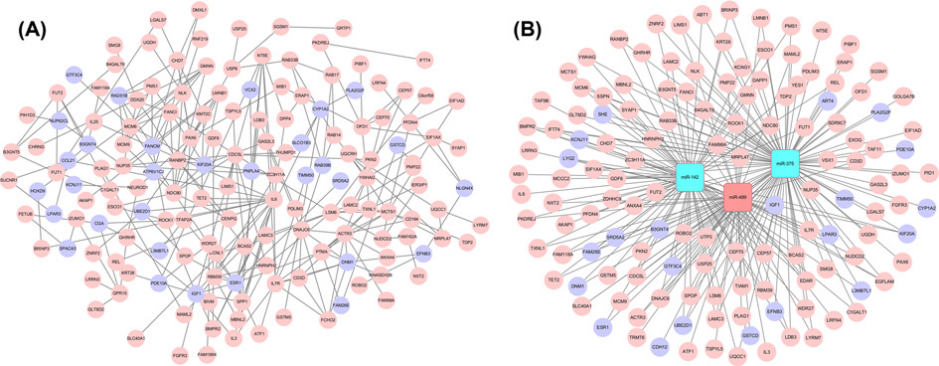
Results of bioinformatics analysis (**A**) Visualization of the PPI network. Different colors distinguish between up-regulated and down-regulated genes. Red nodes are highly expressed genes, while purple nodes are poorly expressed genes. (**B**) The new DEmiRNA-DEGs cross-talk network. The red circle represents the upregulated DEGs, the purple circle represents the downregulated DEGs, the green rectangle represents down-regulated DEmiRNAs, and the red rectangle represents up-regulated DEmiRNAs.

As shown in Figure 1A, 157 DEGs (125 up-regulated and 32 down-regulated genes) containing 157 nodes and 256 edges were present in the PPI network. Since the key nodes could have important roles in biological networks, we calculated all the node values in the PPI network. The top ten candidates were IL6, estrogen receptor 1 (ESR1), ACTR3, NDC80, RANBP2, CDC5L, IGF1, MCTS1, IL7R, and YWHAG.

### Construction of a DEmiRNA–DEG cross-talk network

First, the targets of three DEmiRNAs were predicted from the miRWalk database, after which the target genes and the DEGs were compared, and 136 DE target genes were obtained. Finally, a new MI-related DEmiRNA-DEG regulatory network with 139 nodes and 236 edges was constructed and shown in Figure 1B.

### MiR-489 is up-regulated in H_2_O_2_-induced H9c2 cells

**Figure 2:**
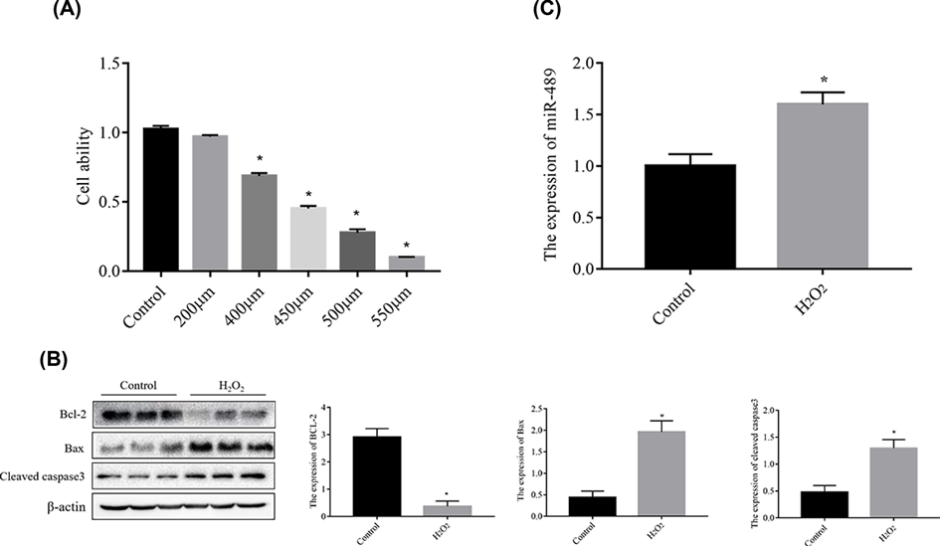
H_2_O_2_ induces H9c2 cell injury and miR-489 is up-regulated in H_2_O_2_- induced H9c2 cardiomyocyte injury (**A**) Cell viability was measured by Cell Counting Kit-8 assays after 0, 100, 200, 400, 450, and 500 μM hydrogen peroxide (H_2_O_2_) treatment for 1 h. (**B**) Expression of apoptosis-associated proteins as detected by Western blot analysis. (**C**) The miR-489 level was detected by RT-qPCR. The data are presented are the means ± standard error of the mean (SEM) (*P<0.05 vs. control; ^#^*P*< 0.05 vs. H_2_O_2_).

The viability of H_2_O_2_-treated H9c2 cells was assessed using a CCK8 kit. Cell viability decreased in dose-dependent manner and reached approximately 50% at 450 μm H_2_O_2_ (Figure 2A). Therefore, we cultured H9c2 cells in 450 μm H_2_O_2_ for 1 h to simulate hypoxia induced by MI *in vitro*. As shown in Figure 2B, the levels of proapoptotic proteins (cleaved caspase3 and Bax) were increased, whereas that of the antiapoptotic protein Bcl-2 was decreased, demonstrating that H_2_O_2_ triggered cell injury. The RT-qPCR results effectively showed miR-489 expression in cardiomyocytes under normal or H_2_O_2_ conditions. As shown in Figure 2C, miR-489 expression was increased in H9c2 cardiomyocytes treated with H_2_O_2_ compared with those cultured under normal conditions (*P*<0.05), suggesting that miR-489 may be involved in mediating H_2_O_2_-induced H9c2 cell damage.

### MiR-489 regulates H_2_O_2_-induced H9c2 cell apoptosis *in vitro*

**Figure 3:**
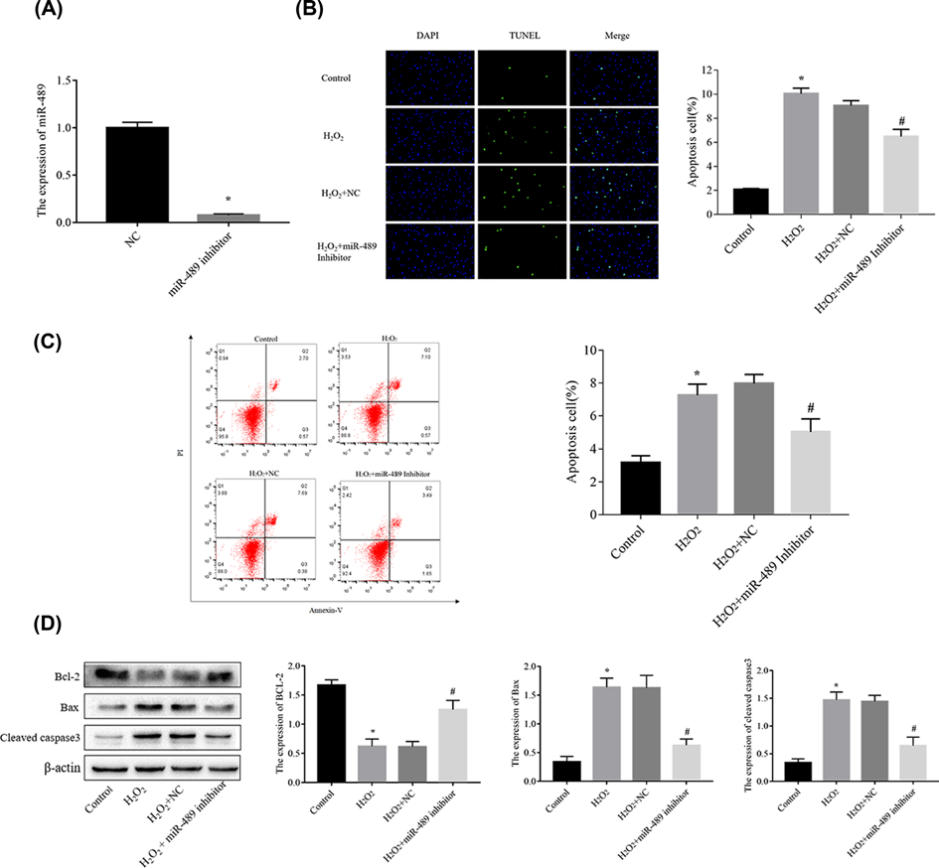
MiR-489 overexpression increases cell apoptosis, while miR-489 inhibition decreases cell apoptosis in H_2_O_2_-treated H9c2 cells (**A**) The miR-489 levels were evaluated by RT-qPCR. (**B**) Cell apoptosis was assessed by TUNEL assay (**C**) Cell apoptosis was evaluated by flow cytometry. (**D**) Expression of apoptosis-associated proteins was analyzed by Western blot analysis. The data are presented are the means ± standard error of the mean (SEM) (*P<0.05 vs. control; ^#^*P*< 0.05 vs. H_2_O_2_).

To further elucidate the effect of miR-489 on H_2_O_2_-induced apoptosis, we silenced miR-489 with miRNA inhibitors. Figure 3A shows that miR-489 expression in the miR- 489 inhibitor group was lower than that observed in the NC group (*P*<0.05). The results of Tunel assays (Figure 3B), flow cytometry (Figure 3C), and Western blot analyses to detect the expression of apoptosis-related proteins (Figure 3D), including Bax, Bcl-2, and cleaved caspase 3 revealed that miR-489 down-regulation decreased H_2_O_2_-induced apoptosis.

### MiR-489 inhibits the expression IGF1 in H_2_O_2_-treated H9c2 cells

**Figure 4:**
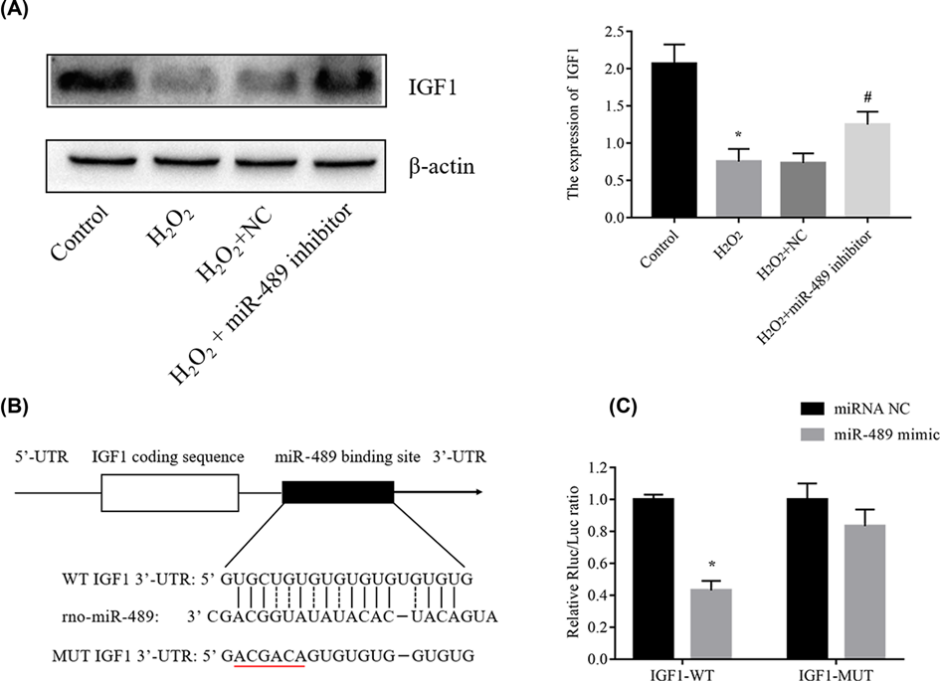
IGF1 is a target of miR-489 (**A**) Schematic diagram illustrating the wild-type (wt) and mutant (mut) 3′-UTR of IGF1 and the corresponding sequence of miR-489. (**B**) H9c2 cardiomyocytes were cotransfected with IGF1-WT or IGF1-Mut and miR-489 mimics or control, after which luciferase activity was assessed using a dual luciferase reporter assay. (**C**) The IGF1 protein levels in cardiomyocytes transfected with miR-489 inhibitor or mimics were detected by Western blot analysis. The data are presented are the means ± standard error of the mean (SEM) (*P<0.05 vs. control; ^#^*P*< 0.05 vs. H_2_O_2_).

To investigate the effect of miR-489 on H_2_O_2_-induced apoptosis, we used miRWalk to predict candidate targets of miR-489 in H9c2 cells. The predicted binding site of the IGF1 3′-UTR and the complementary sequence of miR-489 are shown in Figure 4A. Furthermore, our hypothesis was confirmed by dual luciferase reporter assays. As shown in Figure 4B, the luciferase activity notably decreased after cotransfection with the miR-489 mimic and pGL3-IGF1-wt vector. In contrast, this result was not observed in the group transfected with pGL3-IGF1-mut. Our experimental data showed that miR-489 may participate in H_2_O_2_-induced cardiomyocyte apoptosis by regulating IGF1. In addition, as shown in Figure 4C, the IGF1 protein level decreased after H9c2 cells were treated with H_2_O_2_. In H9c2 cells treated with H_2_O_2_ and the miR-489 inhibitor, the IGF1 level increased compared with that observed in the H_2_O_2_ group. These findings indicate that IGF1 may be a target of miR-489.

## Discussion

An increasing number of basic and clinical studies have uncovered the possible mechanisms underlying the development and progression of MI in the past decades. However, the pathogenesis of MI has not been fully elucidated, and crucial molecular mechanisms involved in MI need to be further investigated. In the present study, mRNA and miRNA expression profile datasets were used to identify DEGs and DEmiRNAs potentially involved in MI. In total, 688 DEGs (including 500 up-regulated and 188 down-regulated mRNAs) and 4 DEMIs (including 1 up-regulated and 3 down-regulated miRNAs) were identified.

In the DEmiRNA-DEG regulatory network, miR-489 may participate in the pathology of MI by regulating IGF1 expression. We used the DAVID database to analyze the function of DEGs, resulting in 10 hub genes with high values (IL6, ESR1, ACTR3, NDC80, RANBP2, CDC5L, IGF1, MCTS1, IL7R, and YWHAG) being identified from the PPI network. These results identified important genes involved in the molecular mechanism of MI initiation and progression, which may be useful for the development of novel treatment strategies.

In the present study, many DE RNAs had a high values in the PPI network, suggesting that they may have important roles in the pathogenesis of MI. Various previous studies have proven that the abnormal expression of ESR1, which had the highest value in our PPI network, was closely associated with cardiovascular diseases. Dysfunction of ESR1 may be one of the causes of acute coronary events [24]. In a previous study, female mice overexpressing ERα underwent LAD coronary ligation followed by reperfusion, and the myocardial fibrosis of the mouse heart muscle was relieved, indicating that ERα could protect against ischemic injury in the hearts of female rodents [25]. A study by Zhai et al. showed that ERα could protect male mice from ischemia/reperfusion (I/R) injury and that ERα knockout may aggravate I/R damage by impeding the calcium influx and disrupting the mitochondrial function [26]. In an I/R injury model of adult female rabbits, acute pretreatment with estrogen or an Erα activator substantially reduced the area of infarction, indicating that estrogen may exert a protective role during I/R through the ER [27]. Furthermore, ERα was shown to play protective roles in the female heart by regulating the activation of p38 MAPK, proapoptotic signaling, and the expression of proinflammatory cytokines [28]. The results of these studies suggested that regulation of ESR1 could be a possible strategy for the treatment of MI.

In the present study, a functional analysis of the 688 identified DEGs showed that they were enriched in various cellular functions, especially histone acetyltransferase activity, the Wnt signaling pathway and other functions.

This finding suggests that the alterations of cardiomyocyte metabolism could have important roles in MI. The reinitiation of blood flow after myocardial ischemia could lead to additional injury to cardiomyocytes, potentially causing MI and heart failure by inducing oxidative stress [29]. The oxidative stress response of cardiomyocytes may be regulated by lysine acetylation. In a rat myocardial I/R model, HDAC6 could deacetylate peroxiredoxin 1 and reduce its activity, increasing ROS production and exacerbating oxidative damage in cardiomyocytes [30].

Moreover, alteration of lysine acetylation was shown to be associated with the development of cardiovascular diseases, including hypertension [31–34], coronary artery disease [35], vascular calcification [36], and heart failure [37]. Additionally, the Wnt pathway inhibitor DKK-1 was shown to relieve atherosclerosis by affecting the proliferation of vascular smooth muscle cells (VSMCs) cultured in hyperlipidemic serum [38]. When N-cadherin was overexpressed, inhibition of classical Wnt signaling could reduce VSMC proliferation by 50% [39]. Kaga et al. observed that intracellular β-catenin was aggregated after the addition of the GSK-3β inhibitor lithium, resulting in decreased cardiomyocyte and vascular endothelial cell apoptosis [40]. In another study, Wnt11 expression was shown to improve the survival of MI and cardiac function by suppressing inflammatory cytokine expression through regulation of NF-κB [41]. Overall, these studies demonstrated that histone acetyltransferase activity and the Wnt signaling pathway have crucial roles in the occurrence and development of MI.

Cytokine–cytokine receptor interactions are associated with health and are important during immunological and inflammatory responses in disease conditions. To establish a new regulatory network related to MI and to discover new molecular mechanisms, we built a DEmiRNA-DEG regulatory network. The network suggested that miR-489 may have important roles by regulating IGF1 in the pathological and physiological processes of MI.

Our experiments also showed that the expression of miR-489 was increased in cardiomyocytes treated with H_2_O_2_, suggesting that miR-489 may be involved in H9c2 cell apoptosis induced by H_2_O_2_. Consequently, inhibition of miR-489 expression may be a possible therapeutic approach to prevent MI. To further explore the functional and molecular mechanisms of miR-489, we used bioinformatics analysis to predict the potential targets of miR-489 and performed luciferase reporter assays to verify the relationship between these molecules.

In our study, IGF1 expression was reduced in H_2_O_2_-induced H9c2 cells, which contrasted with that observed for miR-489. We proved that miR-489 directly binds to the 3′-UTR of IGF1 to inhibit the expression of IGF1. Furthermore, according to previous studies, IGF1 promotes heart survival, which may accelerate protein metabolism, promotes cardiomyocyte growth, and regulates myocardial contraction, while inhibiting apoptosis during cardiac ischemia [42]. Clinical studies have shown that low IGF1 levels may be positively associated with all-cause mortality and recurrence of MI [43]. In addition, IGF1 may have a protective effect on the heart in animal models [44]. Thus, our experimental data are consistent with those of previous studies showing that the reduction of IGF1 may be associated with myocardial injury. In summary, our data showed that miR-489 may be a cardiomyocyte injury factor in MI. First, our results demonstrated that miR-489 expression is increased in H_2_O_2_-treated H9c2 cells. Moreover, miR-489 could regulate apoptosis by targeting IGF1 in myocardial cells. Taken together, these results indicate that miR-489 is a potential therapeutic target for myocardial injury and that IGF1 may be a potential novel myocardial protective factor.

## Supplementary Material

Supplementary Tables S1-S3

## Abbreviations

DAVID: Database for Annotation, Visualization, and Integration Discovery
DEG: differentially expressed gene
DEmiRNA: differentially expressed miRNA
I/R: ischemia/reperfusion
MI: myocardial infarction
PPI: protein–protein interaction
