# Supplementary material for: MiR-489 aggravates H2O2-induced apoptosis of cardiomyocytes via inhibiting IGF1
Source: Biosci Rep. 2020 Sep 16;40(9):BSR20193995. doi: 10.1042/BSR20193995 (PMC7494985; doi:10.1042/BSR20193995)
Supplement: Supplementary Tables S1-S3 [file BSR-2019-3995_supp.pdf]

Supplementary Materials

1. **Supplementary Table S1**

DEmRNA	logFC	P-Value	Regulation
GPR15	3.749676	0.017852	up
PRPH2	3.563579	0.003483	up
ZNF460	-3.23099	0.030764	down
LOC102723377	-3.19464	0.000455	down
ABCB8	3.189761	0.000342	up
LOC105371307	-3.15007	0.00858	down
RNU6ATAC	-3.13955	0.001746	down
C1orf140	-3.08966	0.001683	down
SALL4	-3.08893	0.002086	down
BRINP3	3.082552	0.005634	up

2. Supplementary Table S2

DEmiRNA	ES	P-Value	FDR	Regulation
hsa-miR-1274b	-6.10032	1.06E-09	2.24E-07	Down
hsa-miR-142-3p	-6.13024	8.77E-10	2.24E-07	Down
hsa-miR-375	-6.62421	3.49E-11	2.96E-08	Down
hsa-miR-489	6.124342	9.11E-10	2.24E-07	Up

3. Supplementary Table S3

Term	Count	P Value
**GO BP**		
signal transduction involved in regulation of gene expression	5	0.002734
bleb assembly	4	0.003299
neuron development	6	0.015292
regulation of transcription, DNA-templated	64	0.015715
gamete generation	4	0.015832
positive regulation of transcription from RNA polymerase II promoter	44	0.02219
negative regulation of Wnt signaling pathway	6	0.024837
homophilic cell adhesion via plasma membrane adhesion molecules	11	0.030713
positive regulation of DNA replication	5	0.044464
**GO CC**		
SAGA complex	4	0.013182
Golgi apparatus	40	0.017885
**GO MF**		
histone acetyltransferase activity	6	0.017644
nucleic acid binding	43	0.031575
Cytokine-cytokine receptor interaction	15	0.01294
**KEGG PATHWAY**		
Transcriptional misregulation in cancer	11	0.026393
Glycosphingolipid biosynthesis - lacto and neolacto series	4	0.040473
Intestinal immune network for IgA production	5	0.049705

Supplementary Table S1. The top ten differentially expressed genes.

Supplementary Table S2. Differentially expressed miRNAs.

Supplementary Table S3. Biological function and pathway analysis of DEGs.
